# Effect of fermented *Rhus verniciflua* stokes extract on liver function parameters in healthy Korean adults: a double-blind randomized controlled trial

**DOI:** 10.1186/s13063-021-05656-0

**Published:** 2021-11-22

**Authors:** Jung Hyun Kwak, Hyo-Jeong Lee, Seok-Tae Jeong, Ju Yeon Lee, Minho Lee, Jean Kyung Paik

**Affiliations:** 1 Department of Food and Nutrition, Eulji University, Seongnam, 13135 Gyeonggi-do Republic of Korea; 2 Department of Science in Korean Medicine, College of Korean Medicine, Graduate School, Kyung Hee University, Hoegi-dong, Dongdaemun-gu, Seoul, 02435 Republic of Korea; 3 Fermented Food Science Division, National Institute of Agricultural Sciences, 166, Nongsaengmyeongro, Iseo-myeon, WanjuGun, Jeollabuk-do 55365 Republic of Korea; 4 Department of Food Technology and Services, Eulji University, Seongnam, 13135 Gyeonggi-do Republic of Korea

**Keywords:** Fermented *Rhus verniciflua* Stokes extract, Liver function, Randomized double-blind trial, Korean adult

## Abstract

**Background:**

Fermented *Rhus verniciflua* Stokes extract (FRVE) reported an anti-hepatic lipidemic property mediated by the upregulation of AMP-activated protein kinase (AMPK) in cell and animal models. However, it remains unclear whether there is an effect of FRVE on liver disease-related parameters and serum lipid levels in humans. We investigated the effects of FRVE intake for 12 weeks on liver disease-related parameters and serum lipid profiles in Korean adults.

**Methods:**

A randomized, double-blind, placebo-controlled study was conducted among 79 subjects. An aqueous extract of fermented *Rhus verniciflua* Stokes that was filtered and fermented was prepared. For 12 weeks, the test group (*n* = 39) consumed two capsules of FRVE (main components: fustin 129 mg and fisetin 59 mg) once daily. The control group (*n* = 40) consumed two placebo pills (main component: lactose 627.0 mg) once daily. A 1:1 randomization of control and test was performed using computer-generated randomization. Both before and after FRVE intake, anthropometric parameters, liver function-related parameters, and clinical laboratory parameters were measured. The effects between the test and control groups were compared using the Mann-Whitney *U* test and independent *t*-test, and the difference between baseline and follow-up values was compared using Wilcoxon rank-sum test and paired *t*-test.

**Results:**

There was no significant difference when comparing the change values of liver disease-related parameters and serum lipid profiles in between groups.

**Conclusions:**

In our study, we did not confirm the significance in liver function parameters and serum lipid profiles.

**Trial registration:**

The study protocol was registered in the Clinical Research Information Service (CRIS: https://cris.nih.go.kr/cris/index.jsp) under number KCT0005687. Registered on 2 December 2020

**Supplementary Information:**

The online version contains supplementary material available at 10.1186/s13063-021-05656-0.

## Background

Based on the Korea National Health and Nutrition Survey data from 1998 to 2017, a recent study reported that the prevalence of nonalcoholic fatty liver disease (NAFLD) is increasing rapidly, especially in men, estimating that NAFLD would reach 44% by 2035 [1]. Subjects with NAFLD have a higher mortality rate from liver diseases or other causes, and the risk of cardiovascular disease and chronic kidney disease is also increased compared to that in subjects without NAFLD [2, 3]. Therefore, prevention and treatment in subjects with NAFLD are important. But, since there is no registered drug for the treatment of NAFLD, diet and exercise are considered very important treatment methods [4].

*Rhus verniciflua* Stokes (RVS) is known by the common name lacquer tree, which is an Asian tree species of the *Anacardiaceae* family and cultivated in regions of China, Japan, and Korea [5]. The sap that comes out of a wound on the bark of a lacquer tree is called lacquer, and lacquer has been widely used for food and medicine since ancient times. The lacquer sap contains various active ingredients such as urushiol, fisetin, fustin, and butein, but urushiol is an allergic ingredient and causes connection dermatitis, so the range of use of lacquer was very limited [6]. However, in Korea, regulations on the use of urshiol-removed lacquer tree extract have been relaxed since 2004. So, various methods to remove urushiol have been developed and used [7].

Fermented *Rhus verniciflua* Stokes extract (FRVE) is a food that is free of urushiol allergens and contains two kinds of flavonoids such as fustin and fisetin. FRVE has been reported to have anti-hepatic lipidemic effects through the activation of AMPK [7]. Furthermore, Lee et al. reported in an animal model, in which the formation of a nonalcoholic fatty liver was induced by a high-fat diet, that ingestion of FRVE reduced liver-related parameters, such as alanine aminotransferase (ALT) and aspartate aminotransferase (AST), and reduced lipid-related parameters such as total cholesterol (TC) and triglycerides (TG) [8]. However, the effect of FRVE intake on liver function parameters has not been identified in humans. Therefore, we investigated the effect of intake of FRVE for 12 weeks on liver disease-related parameters and lipid profiles in Korean adults.

## Methods

### Preparation of fermented *Rhus verniciflua* Stokes extracts

For the FRVE used in this study, 300 kg of lacquer bark (Okcheonsan, Chungbuk, Korea) was cut into 2 × 2 cm pieces and placed in a bag that allows moisture to pass through. In order to absorb moisture in the bark of the sumac tree, it was immersed in water for 1 day and drained for 1 day. The lacquer bark was removed and transferred to a 5-L mushroom cultivation bag, and a filter was attached and sterilized at 121°C for 100 min. Then, the FRVE was prepared by mixing and inoculating a long-lived mushroom starter (*Fomitella fraxinea*) prepared according to the method of Choi et al. [9], culturing at 21°C for 30 days, and drying it with hot air at 50°C. Ten times distilled water was added to 100 g of fermented *Rhus veniciflua* Stokes (FRV) ground with a mixer. Its weight was measured, and it was put into an autoclave and extracted for 8 h at an interval of 1 h at 80–120 °C. Thereafter, water was added to correct the amount of evaporated moisture and adjusted to the same weight. The crude extract was centrifuged (4 °C, 12,000×g, 30 min) and filtered (Filter paper No. 2, Whatman™, GE Healthcare Co., Buckinghamshire, UK). The solid content was obtained by drying 200 mL of the filtered FRVE at 105 °C and was calculated as the solid content for 100 g of FRVE. We used the FRVE samples that went through the above standardization process provided by the Rural Development Administration.

### Study subjects

Subjects were recruited from June to October 2020. Subjects were recruited from local advertisements in the Eulji University Seongnam Campus area. Clinical trial recruitment posters contained brief information about the trial and were posted in Eulji University Seongnam Campus area after institutional review board approval. Subjects included were (1) male or female subjects with age ≥ 20 years, (2) subjects without any diseases, and (3) subjects who agreed to participate in the clinical trial and signed the informed consent form. Subjects were excluded if they had any diagnosis of renal disease, liver disease, cardiovascular disease, and cancer or consumed any disease-related drugs.

This study was conducted as a double-blind randomized controlled design. We explained this study to the subjects and obtained written consent before initiating the study. The test and placebo capsules were provided and blinded by the Rural Development Administration (Jeollabuk-do, Republic of Korea). The 500-mg test capsule is composed of FRVE 400 mg, crystalline cellulose 75 mg, calcium carboxymethyl cellulose 15 mg, silica 5 mg, and magnesium stearate 5mg. The 500-mg placebo capsule is composed of lactose powder 313.5 mg, caramel color 75 mg, gardenia blue color 1.5 mg, crystalline cellulose 105 mg, and magnesium stearate 5 mg. The randomization sequence was generated by the Rural Development Administration using a computer-generated randomization (control:test = 1:1) (https://www.randomizer.org/). To maintain the blinding, the appearance of the test and placebo product was identical. For 12 weeks, the test group (*n* = 39) consumed two capsules of FRVE (main components: fustin 129 mg and fisetin 59 mg) once per day. The control group (*n* = 40) consumed two placebo pills (main components: lactose 627.0 mg) once per day. Subjects visited the Clinical Nutrition Laboratory located in Eulji University 3 times during the study period. Scheduled assessments were conducted at screening and baseline (week 0) and at the end of weeks 6 and 12. Participants were encouraged to maintain their usual lifestyle and dietary habits and to keep a food diary. Compliance was assessed by counting the remaining packs at 12 weeks, and if more than 80% of the packs were consumed, compliance was considered acceptable.

### Human dose calculation

In the experimental study, the conditions for using a dose of 2000 mg/kg or less of FRVE were established based on the results of the toxicity test conducted by the ChemOn Preclinical Research Center (Gyeonggi-do, Korea), and the effective concentration of FRVE in this study was derived based on a previous study [8, 10, 11].

### Sample size

To calculate the number of clinical trial subjects in this study, the average value of 32.7 for those with 25 IU/L or more among the general AST data presented by Kim et al.’s study was referenced [12]. Since a 10% reduction in this average value can be regarded as an effective reduction value, the average difference between the treatment group and the control group was assumed to be 3.27, and the standard deviation was assumed to be 4.0. Assuming a significance level of 5% and power of 80%, *Z*_1*α*/2_ = 1.96 (two-tailed test), *Z*_1-*β*_ = 0.845, *D*_t_−*D*_c_ = 3.27, and *σ* = 4.0 were substituted into the formula. The number of subjects was calculated considering the dropout rate of 20% and the compliance rate of 80%. The number of subjects in each group was 35 or more, and a total of 79 subjects were recruited.

### Anthropometric parameters and blood collection

Body weight and height were measured in the morning in unclothed subjects without shoes to calculate the body mass index (BMI; kg/m^2^). Blood pressure (BP) was measured at baseline and at the 12-week follow-up visits using a sphygmomanometer (Omron HEM-7120, OMRON Co., Ltd, Japan). We obtained three BP measurements at each visit, and differences among the three systolic blood pressure (SBP) readings were always <5 mmHg. Participants were instructed not to smoke or drink alcohol for at least 30 min before measuring BP. Patients were also instructed to undergo a 12-h fasting period before the initial blood draw and before the follow-up visits. Venous blood specimens were collected in EDTA-treated plain tubes and then centrifuged to produce plasma or serum, which was subsequently stored at −70 °C until analysis.

### Liver function-related parameters and lipid profile

The serum levels of AST, ALT, and gamma-glutamyl transferase (GGT) were measured using aspartate aminotransferase, alanine aminotransferase, and ɣ-glutamyltransferase kits (Roche, Germany) and a Cobas 800 analyzer (Roche, Germany), according to the manufacturer’s instructions. Alkaline phosphatase levels were measured using an ALP2 kit (Roche, Germany) and a Cobas 800 analyzer. Fasting serum TC, high-density lipoprotein (HDL) cholesterol, and TG levels were measured using commercially available kits and a Cobas 8000 analyzer. Low-density lipoprotein (LDL) cholesterol was indirectly estimated in participants with serum triglyceride levels < 400 mg/dL using the Friedewald formula: LDL cholesterol = TC − (HDL cholesterol + (TG/5)).

### Laboratory blood parameters

Complete blood count (CBC), white blood cell (WBC) count (10^3^/μL), red blood cell (RBC) count (10^6^/μL), platelet (PLT) count (10^3^/μL), hematocrit (Hct) (%), and hemoglobin (Hb) level (g/dL) were measured using commercially available kits and the Sysmex analyzer (Sysmex, Japan). The levels of glucose, insulin, free fatty acid, blood urea nitrogen, and creatinine were measured using commercially available kits and the Cobas 8000 analyzer.

### Assessment of food intake

The diet information was obtained using a 24-h recall method. All subjects received written and oral instructions by a dietitian on how to complete the 3-day (2 weekdays and 1 weekend day) dietary records. On the recording sheet, subjects wrote the kinds and amounts of food they consumed. Dietary energy values and nutrient content from all complete 3-day dietary records were calculated using the Computer-Aided Nutritional Analysis Program (CAN-Pro 5.0, Korean Nutrition Society, Seoul, Korea).

### Data analysis

Statistical analysis was performed using Statistical Package for the Social Sciences (SPSS), version 20.0 for Windows (SPSS, Chicago, IL, USA). Skewed variables were logarithmically transformed for the statistical analyses. Categorized variables were tested using the chi-square test. Mann-Whitney *U* test (nonparametric test) and independent *t*-test were used for comparing the effects between the test and control groups. Wilcoxon rank-sum test (nonparametric test) and paired *t*-test were used for comparing the baseline and follow-up values. In groups with < 20 subjects, a nonparametric test was used. For descriptive purposes, mean values are presented using untransformed values. Results are expressed as mean ± standard error (SE). A two-tailed *p*-value < 0.05 was considered statistically significant. We used AST as the primary efficacy outcome, and other liver indicators such as ALT and GGT, and lipid parameters were used as secondary efficacy outcomes.

## Results

### Characteristics of study participants

Altogether, among the enrolled subjects (*n* = 79, test = 39, and control = 40), 27 dropped out due to refusal to revisit amidst the coronavirus disease pandemic (*n* = 10), personal reasons including employment commitments and relocating (*n* = 5), and suboptimal compliance with FRVE intake (*n* = 12). Finally, 52 subjects (test = 27, control = 25) completed the study (CONSORT 2010 Flow Diagram).

Table 1 shows the clinical characteristics of participants in the control and the test group at baseline. There were no significant differences between the two groups in the distribution of age, sex, height, weight, SBP, diastolic blood pressure (DBP), and blood parameters.

**Table 1:** Comparison of the clinical characteristics of the subjects at baseline

Characteristic	Group (total = 52)		
	Control (***n*** = 25)	FRVE (***n*** = 27)	***p***
Sex, no. (%) of participant			0.893
Men	5 (20.0)	5 (18.5)	
Women	20 (80.0)	22 (81.5)	
Age (years)	33.24 (±3.06)	33.07 (±2.92)	0.969
Height (cm)	162.55 (±1.58)	163.37 (±1.19)	0.679
Weight (kg)	59.36 (±2.66)	63.69 (±2.06)	0.201
Waist (cm)	74.61 (±2.36)	79.87 (±2.35)	0.121
Hip (cm)	94.66 (±1.62)	95.67 (±1.46)	0.641
SBP (mmHg)	111.48 (±2.82)	111.93 (±2.45)	0.905
DBP (mmHg)	68.40 (±2.20)	69.96 (±2.08)	0.608
Blood parameters			
Glucose (mg/dL)	92.64 (±2.32)	89.78 (±1.67)	0.337
Insulin (uU/mL)	10.99 (±2.89)	16.44 (±3.32)	0.074
Albumin (g/dL)	4.94 (±0.06)	4.94 (±0.04)	0.964
WBC (mm^3^)	6.31 (±0.30)	6.37 (±0.25)	0.885
RBC (mm^3^)	4.60 (±0.09)	4.46 (±0.07)	0.205
Hb (g/dL)	13.82 (±0.25)	13.67 (±0.22)	0.657
HCT (%)	41.09 (±0.62)	40.59 (±0.57)	0.555
PLT (10^3^/uL)	258.36 (±11.88)	235.67 (±11.64)	0.179
AST (IU/L)	20.48 (±1.44)	20.15 (±1.12)	0.995
ALT (IU/L)	17.67 (±2.71)	16.63 (±1.62)	0.949
AST/ALT ratio	1.44 (±0.10)	1.40 (±0.09)	0.765
GGT (mg/dL)	18.32 (±2.02)	21.59 (±3.06)	0.384
ALP (IU/L)	64.56 (±2.92)	66.00 (±4.21)	0.783
TC (mg/dL)	190.84 (±5.08)	179.44 (±5.69)	0.144
TG (mg/dL)	125.76 (±23.55)	138.74 (±22.23)	0.589
HDL-C (mg/dL)	60.44 (±2.93)	62.59 (±3.48)	0.641
LDL-C (mg/dL)	105.25 (±5.39)	92.26 (±5.61)	0.102
MDA (pmol/mL)	321.30 (±70.57)	225.69 (±40.30)	0.373
Adiponectin (ug/uL)	6.89 (±0.53)	6.57 (±0.40)	0.628
Oxi-LDL (U/L)	40,718.78 (±3307.54)	35,885.09 (±2434.90)	0.240

Independent *t*-test was used for continuous variables (mean ± SE) and chi-square test was used for categorical variables (*n*, %)

*AST* aspartate aminotransferase, *ALT* alanine aminotransferase, *ALP* alkaline phosphatase, *DBP* diastolic blood pressure, *GGT* ɣ-glutamyl transferase, *HCT* hematocrit, *Hb* hemoglobin, *HDL-C* high-density lipoprotein cholesterol, *LDL-C* low-density lipoprotein cholesterol, *MDA* malondialdehyde, *PLT* platelet, *RBC* red blood cells, *SBP* systolic blood pressure, *TC* total cholesterol, *TG* triglycerides, *WBC* white blood cells

### Effect of FRVE supplementation on liver function and lipid metabolism parameters

Table 2 shows the effect of FRVE supplementation on liver function and lipid metabolism parameters. The net changes in liver function and lipid metabolism parameters were not significantly different between the two groups.

**Table 2:** Liver function-related parameters and serum lipid levels before and 12 weeks after consuming FRVE

	Group (total = 52)						
	Control (***n*** = 25)			FRVE (***n*** = 27)			
	0 weeks	12 weeks	***p***^**a**^	0 weeks	12 weeks	***p***^**a**^	***p***^**b**^
AST (IU/L)*	20.48 (± 1.44)	19.88 (± 1.24)	0.778	20.15 (± 1.12)	20.85 (±1.75)	0.836	
**△ change**	−0.60 (± 1.38)			0.70 (±0.96)			0.436
ALT (IU/L)*	17.67 (± 2.71)	17.25 (± 1.96)	0.500	16.63 (± 1.62)	16.89 (±1.90)	0.928	
**△ change**	−0.42 (± 1.88)			0.26 (±1.31)			0.765
**AST/ALT ratio**	1.44 (± 0.10)	1.36 (± 0.10)	0.095	1.40 (± 0.09)	1.38 (±0.08)	0.832	
**△ change**	−0.09 (± 0.05)			−0.02 (±0.09)			0.523
GGT (mg/dL)*	18.32 (± 2.02)	17.48 (± 1.99)	0.241	21.59 (± 3.06)	19.44 (±2.88)	0.063	
**△ change**	−0.84 (± 0.70)			−2.15 (±1.11)			0.331
**ALP (IU/L)**	64.56 (± 2.92)	63.08 (± 2.50)	0.289	66.00 (± 4.21)	65.31 (±4.28)	0.608	
**△ change**	−1.48 (± 1.36)			−0.63 (±1.21)			0.642
**TC (mg/dL)**	190.84 (± 5.07)	187.20 (± 4.93)	0.231	179.44 (± 5.69)	176.56 (±5.89)	0.377	
**△ change**	−3.64 (± 2.96)			−2.89 (±3.22)			0.865
TG (mg/dL)*	125.76(± 23.55)	97.68 (± 11.12)	0.073	138.74 (± 22.23)	110.70 (±17.26)	0.046	
**△ change**	−28.08 (± 13.64)			−28.04 (±14.20)			0.998
**HDL-C (mg/dL)**	60.44 (± 2.93)	62.32 (± 2.44)	0.184	62.59 (± 3.48)	62.52 (±3.23)	0.957	
**△ change**	1.88 (± 1.38)			−0.07 (±1.37)			0.320
**LDL-C (mg/dL)**	105.25 (± 5.39)	105.34 (± 5.01)	0.976	92.26 (± 5.61)	95.73 (±5.71)	0.306	
**△ change**	0.10 (± 3.21)			3.47 (±3.32)			0.469

Data are presented as mean ± SE

*AST* aspartate aminotransferase, *ALT* alanine aminotransferase, *ALP* alkaline phosphatase, *GGT* ɣ-glutamyl transferase, *HDL-C* high-density lipoprotein cholesterol, *LDL-C* low-density lipoprotein cholesterol, *TC* total cholesterol, *TG* triglycerides

^*^Tested using logarithmic transformation

^a^*p*-values were calculated using paired *t*-test when comparing the baseline values (0 weeks)

^b^*p*-values were calculated using the independent *t*-test comparing the “change” values

When analyzing liver function and lipid metabolism parameters by sex, no significant differences were found in the liver function and lipid metabolism parameters (Tables 3 and 4).

**Table 3:** Liver function-related parameters and serum lipids before and 12 weeks after consuming FRVE among men

	Group (total = 10)						
	Control (***n*** = 5)			FRVE (***n*** = 5)			
	0 weeks	12 weeks	***p***^**a**^	0 weeks	12 weeks	***p***^**a**^	***p***^**b**^
AST (IU/L)*	22.40 (± 2.98)	24.60 (± 4.40)	0.273	25.80 (± 3.14)	30.00 (±7.33)	0.465	
**△ change**	2.20 (± 2.44)			4.20 (±4.35)			0.834
ALT (IU/L)*	25.20 (± 6.93)	26.20 (± 6.03)	0.581	29.00 (± 2.66)	28.80 (±6.11)	0.684	
**△ change**	1.00 (± 1.70)			−0.20 (±4.75)			0.461
**AST/ALT ratio**	1.03 (± 0.14)	0.99 (± 0.11)	0.500	0.90 (± 0.09)	1.02 (±0.04)	0.225	
**△ change**	−0.04 (± 0.06)			0.12 (±0.11)			0.175
GGT (mg/dL)*	20.40 (± 3.04)	20.60 (± 2.16)	0.891	35.00 (± 10.89)	27.60 (±7.81)	0.068	
**△ change**	0.20 (± 2.58)			−7.40 (±3.79)			0.169
**ALP**	56.60 (± 3.57)	56.00 (± 2.59)	0.892	64.00 (± 4.16)	62.40 (±3.44)	0.461	
**△ change**	−0.60 (± 1.69)			−1.60 (±3.53)			0.916
**TC (mg/dL)**	194.20 (± 12.87)	191.60 (± 12.50)	0.498	176.80 (± 11.32)	169.40 (±15.50)	0.225	
**△ change**	−2.60 (± 10.67)			−7.40 (±6.61)			0.832
TG (mg/dL)*	152.20 (± 72.55)	113.40 (± 24.49)	0.893	275.40 (± 58.19)	144.40 (±37.27)	0.043	
**△ change**	−38.8 (± 50.50)			−131.0 (±43.32)			0.076
**HDL-C (mg/dL)**	51.00 (± 3.82)	56.20 (± 4.75)	0.223	43.60 (± 5.56)	47.60 (±3.47)	0.138	
**△ change**	5.20 (± 3.18)			4.00 (±2.35)			0.916
**LDL-C (mg/dL)**	112.76 (± 20.55)	112.72 (± 12.61)	0.893	78.12 (± 9.75)	92.92 (±13.76)	0.080	
**△ change**	−0.04 (± 10.47)			14.80 (±7.19)			0.175

Data are presented as mean ± SE. Data are presented as mean ± SE

*AST* aspartate aminotransferase, *ALT* alanine aminotransferase, *ALP* alkaline phosphatase, *GGT* ɣ-glutamyl transferase, *HDL-C* high-density lipoprotein cholesterol, *LDL-C* low-density lipoprotein cholesterol, *TC* total cholesterol, *TG* triglycerides

*Tested using logarithmic transformation

^a^*p*-values were calculated using the Wilcoxon test comparing the baseline values (0 weeks)

^b^*p*-values were calculated using the Mann-Whitney test when comparing the “change” values

**Table 4:** Liver function-related parameters and serum lipids before and 12 weeks after consuming FRVE among women

	Group (total = 42)						
	Control (***n*** = 20)			FRVE (***n*** = 22)			
	0 weeks	12 weeks	***p***^**a**^	0 weeks	12 weeks	***p***^**a**^	***p***^**b**^
AST (IU/L)*	20.00 (± 1.66)	18.70 (± 1.02)	0.581	18.86 (± 1.03)	18.77 (±1.10)	0.776	
**△ change**	−1.30 (± 1.60)			−0.09 (±0.64)			0.699
ALT (IU/L)*	15.68 (± 2.83)	14.89 (± 1.61)	0.669	13.82 (± 1.29)	14.18 (±1.41)	0.798	
**△ change**	−0.79 (± 2.34)			0.36 (±1.28)			0.818
**AST/ALT ratio**	1.55 (± 0.11)	1.45 (± 0.12)	0.119	1.52 (± 0.10)	1.46 (±0.09)	0.646	
**△ change**	−0.10 (± 0.06)			−0.05 (±0.11)			0.235
GGT (mg/dL)*	17.80 (± 2.42)	16.70 (± 2.42)	0.023	18.55 (± 2.59)	17.59 (±3.01)	0.112	
**△ change**	−1.10 (± 0.64)			−0.95 (±0.93)			0.136
**ALP**	66.55 (± 3.42)	64.85 (± 2.95)	0.320	66.45 (± 5.11)	66.05 (±5.21)	0.755	
**△ change**	−1.70 (± 1.67)			−0.41 (±1.30)			0.805
**TC (mg/dL)**	190.00 (± 5.64)	186.10 (± 5.46)	0.180	180.05 (± 6.60)	178.18 (±6.45)	0.618	
**△ change**	−3.90 (± 2.80)			−1.86 (±3.68)			0.667
TG (mg/dL)*	119.15 (± 24.23)	93.75 (± 12.64)	0.054	107.68 (± 18.88)	103.05 (±19.43)	0.421	
**△ change**	−25.40 (± 12.56)			−4.64 (±9.17)			0.203
**HDL-C (mg/dL)**	62.80 (± 3.36)	63.85 (± 2.76)	0.494	66.91 (± 3.51)	65.91 (±3.52)	0.525	
**△ change**	1.05 (± 1.51)			−1.00 (±1.55)			0.769
**LDL-C (mg/dL)**	103.37 (± 4.77)	103.50 (± 5.50)	0.968	95.63 (± 6.42)	96.40 (±6.43)	0.831	
**△ change**	0.13 (± 3.24)			0.77 (±3.56)			0.276

Data are presented as mean ± SE

*AST* aspartate aminotransferase, *ALT* alanine aminotransferase, *ALP* alkaline phosphatase, *GGT* ɣ-glutamyl transferase, *HDL-C* high-density lipoprotein cholesterol, *LDL-C* low-density lipoprotein cholesterol, *TC* total cholesterol, *TG* triglycerides

*Tested using logarithmic transformation

^a^*p*-values were calculated using the Wilcoxon test comparing the baseline values (0 weeks)

^b^*p*-values were calculated using the Mann-Whitney test when comparing the “change” values

### Effect of FRVE supplementation on laboratory blood parameters

Table 5 shows the effect of FRVE supplementation on laboratory blood parameters. PLT significantly increased in the test group, and the changes in PLT were significantly different between the two groups.

**Table 5:** Laboratory blood parameters before and 12 weeks after consuming FRVE

	Group (total = 52)						
	Control (***n***= 25)			FRVE (***n***= 27)			
	0 weeks	12 weeks	***p***^**a**^	0 weeks	12 weeks	***p***^**a**^	***p***^**b**^
Glucose (mg/dL)*	92.64 (± 2.32)	97.04 (± 7.46)	0.712	89.78 (± 1.67)	87.41 (±2.32)	0.312	
**△ change**	4.40 (± 6.37)			−2.37 (±2.68)			0.319
Insulin (μU/mL)*	10.99 (± 2.89)	9.78 (± 1.61)	0.822	16.44 (± 3.32)	10.62 (±1.72)	0.046	
**△ change**	−1.22 (± 2.03)			−5.81 (±3.25)			0.245
**HOMA-IR**	2.52 (± 0.64)	2.49 (± 0.49)	0.948	3.62 (± 0.70)	2.22 (±0.32)	0.043	
**△ change**	−0.03 (± 0.51)			−1.41 (±0.66)			0.112
**FFA**	604.20 (± 74.01)	547.96 (± 57.26)	0.440	581.19 (± 53.09)	556.26 (±52.13)	0.681	
**△ change**	−56.24 (± 71.57)			−24.93 (±59.88)			0.737
**BUN (mg/dL)**	11.71 (± 0.55)	11.75 (± 0.76)	0.942	11.84 (± 0.58)	11.83 (±0.56)	0.991	
**△ change**	0.04 (± 0.54)			−0.01 (±0.66)			0.956
**Creatinine (mg/dL)**	0.80 (± 0.04)	0.78 (± 0.03)	0.307	0.76 (± 0.02)	0.73 (±0.02)	0.015	
**△ change**	−0.02 (± 0.02)			−0.03 (±0.01)			0.398
**Albumin (g/dL)**	4.94 (± 0.06)	4.82 (± 0.05)	0.047	4.94 (± 0.04)	4.87 (±0.05)	0.191	
**△ change**	−0.12 (± 0.06)			−0.07 (±0.06)			0.567
**WBC (mm**^**3**^**)**	6.31 (± 0.30)	6.60 (± 0.41)	0.452	6.37 (± 0.25)	6.29 (±0.24)	0.733	
**△ change**	0.29 (± 0.38)			−0.08 (±0.23)			0.400
**RBC (mm**^**3**^**)**	4.60 (± 0.09)	4.57 (± 0.09)	0.507	4.46 (± 0.07)	4.35 (±0.08)	0.007	
**△ change**	−0.03 (± 0.04)			−0.11 (±0.04)			0.166
**HGB (g/dL)**	13.82 (± 0.24)	13.69 (± 0.24)	0.270	13.67 (± 0.22)	13.41 (±0.23)	0.028	
**△ change**	−0.13 (± 0.12)			−0.27 (±0.11)			0.415
**HCT (%)**	41.09 (± 0.62)	41.01 (± 0.64)	0.836	40.59 (± 0.57)	39.79 (±0.56)	0.016	
**△ change**	−0.08 (± 0.38)			−0.80 (±0.31)			0.145
**PLT (10**^**3**^**/μL)**	258.36 (± 11.88)	247.76 (± 13.20)	0.546	235.67 (± 11.64)	250.81 (±12.08)	0.014	
**△ change**	−10.60 (± 9.76)			15.15 (±5.77)			0.025

Data are presented as mean ± SE

*BUN* blood urea nitrogen, *FFA* free fatty acids, *HOMA-IR* homeostasis model assessment, *Hb* hemoglobin, *HCT* hematocrit, *PLT* platelet, *RBC* red blood cells, *WBC* white blood cells

*Tested using logarithmic transformation

^a^*p*-values were calculated using the paired *t*-test when comparing the baseline values (0 weeks)

^b^*p*-values were calculated using the independent *t*-test when comparing the “change” values

### Effect of FRVE supplementation on dietary parameters

Table 6 shows the effect of FRVE supplementation on dietary parameters. The net changes in dietary parameters were not significantly different between the two groups.

**Table 6:** Dietary parameters before and 12 weeks after consuming FRVE

	Group (total = 52)						
	Control (***n*** = 25)			FRVE (***n*** = 27)			
	0 weeks	12 weeks	***p***^**a**^	0 weeks	12 weeks	***p***^**a**^	***p***^**b**^
**Energy intake (kcal)**	1625.72 (± 89.40)	1614.61 (± 103.00)	0.919	1621.91 (± 94.29)	1562.36 (±94.56)	0.635	
**△ change**	−11.11 (± 108.50)			−59.54 (± 124.02)			0.772
**Carbohydrate (g)**	225.22 (± 13.62)	205.70 (± 11.90)	0.091	217.18 (± 12.14)	216.01 (±13.94)	0.941	
**△ change**	−19.52 (± 11.07)			−1.17 (± 15.76)			0.352
**Protein (g)**	64.71 (± 6.40)	70.37 (± 5.79)	0.377	64.15 (± 4.97)	62.65 (±6.24)	0.855	
**△ change**	5.66 (± 6.29)			−1.50 (± 8.15)			0.495
**Fat (g)**	45.39 (± 3.78)	54.34 (± 6.06)	0.198	52.12 (± 5.63)	48.28 (±4.51)	0.585	
**△ change**	8.95 (± 6.75)			−3.84 (± 6.94)			0.194
**Fiber (g)**	15.49 (± 1.45)	14.26 (± 1.40)	0.305	15.42 (± 1.07)	16.36 (±1.19)	0.411	
**△ change**	−1.24 (± 1.18)			0.93 (± 1.12)			0.188
Vitamin A (μg RAE)*	400.39 (± 70.07)	306.67 (± 34.13)	0.207	347.65 (± 47.74)	319.64 (±46.08)	0.575	
**△ change**	−93.72 (± 74.16)			−28.01 (± 75.81)			0.539
Vitamin D (μg)*	4.79 (± 1.24)	2.44 (± 0.53)	0.037	6.52 (± 2.05)	4.76 (±2.45)	0.101	
**△ change**	−2.36 (± 1.17)			−1.76 (± 3.19)			0.866
Vitamin C (mg)*	63.89 (± 13.23)	63.26 (± 11.42)	0.876	62.42 (± 9.56)	60.32 (±11.52)	0.444	
**△ change**	−0.63 (± 16.22)			−2.10 (± 15.07)			0.947
Calcium (mg)*	512.95 (± 84.25)	395.18 (± 57.18)	0.214	443.91 (± 54.91)	386.75 (±60.78)	0.219	
**△ change**	−117.77 (± 86.79)			−57.16 (± 85.10)			0.620
**Phosphate (mg)**	974.40 (± 90.37)	986.10 (± 74.54)	0.903	962.99 (± 67.42)	913.07 (±82.33)	0.625	
**△ change**	11.70 (± 95.07)			−49.92 (± 100.96)			0.660
**Sodium (mg)**	2648.13 (± 211.47)	3385.61 (± 272.91)	0.036	2937.52 (± 247.34)	3101.09 (±298.88)	0.668	
**△ change**	737.49 (± 331.77)			163.57 (± 376.46)			0.261
**Cholesterol (mg)**	382.76 (± 56.06)	305.93 (± 51.42)	0.173	295.72 (± 45.19)	337.85 (±59.57)	0.523	
**△ change**	−76.83 (± 54.74)			42.13 (± 65.06)			0.171

Data are presented as mean ± SE

*Tested using logarithmic transformation

^a^*p*-values were calculated using the paired *t*-test when comparing baseline values (0 weeks)

^b^*p*-values were calculated using the independent *t*-test when comparing the “change” values

## Discussion

In our study, there was no significant difference when comparing the change values of liver disease-related parameters and serum lipid profiles in between groups.

*Rhus verniciflua* Stokes extract (RVSE) has been reported to have various functions including anticancer [13], antioxidant [14], and anti-inflammatory [15] properties, but its use is limited due to the presence of urushiol, an allergen in RVSE. Therefore, various methods for removing urushiol have been studied and developed [11, 16]. In our study, urushiol-free FRVE was used according to the method used by Choi et al. [9].

The Korean Ministry of Food and Drug Safety approved daily intake of 1 g FRVE powder (fustin 57mg/g), as “it can help male climacteric health” [17]. On the other hand, there are no clinical studies on liver function. Lee et al. reported that FRVE has an anti-hepatic lipidemic effect by upregulating AMPK in cell and animal models [8, 11]. In particular, they confirmed in an animal model that the levels of ALT, AST, TC, and TG decreased when animals with nonalcoholic fatty liver ingested FRVE [8]. ALT and AST are blood indicators that increase during liver damage and are important parameters that can predict the state of liver diseases such as hepatocellular disease or hepatitis [18]. GGT has also been reported as an indicator of internal organ fat, fatty liver [19, 20], and IR [21]. The liver is a major organ for the synthesis and metabolism of lipoproteins. If there is a dysfunction in the liver, dyslipidemia may occur, in which HDL cholesterol is lowered and triglycerides and total cholesterol are elevated [22, 23]. In particular, NAFLD is often accompanied by IR [24], obesity [25], and diabetes [26]; therefore, changes in serum lipid levels and IR are also used as auxiliary indicators to measure liver health and function. Hyperinsulinemia caused by IR induces decreased glycogen synthesis, increased fatty acid uptake in the liver, altered TG transport, inhibited fatty acid oxidation, and increased lipolysis in peripheral tissues, resulting in the accumulation of TG in the liver [27]. Therefore, the reduction of IR will help prevent and treat NAFLD. However, since there was no significant difference in the change value of IR between the test and control groups in our study, so additional confirmation through further study is warranted.

Functions of FRVE reported through other studies include anti-obesity [28], anti-cancer [29, 30], and anti-inflammatory properties [5]. One animal study reported the anti-obesity effect of *R. verniciflua* leaf extract [28], but we could not confirm the effect of weight loss in humans. This is considered to be due to the large proportion of nonobese people in our study group. Previous clinical studies investigating the benefits of FRVE or RVSE only confirmed the effect on survival rate and clinical symptoms in patients with colon cancer [29, 30]. In general, the effects of functional foods may be more effective in those who are on the border of disease or have diseases than in healthy people. Since our study was conducted on healthy adults, it may not have been possible to confirm the change in indicators related to liver function. In future studies, it will be necessary to verify the efficacy of FRVE in subjects with high liver-related parameters.

This study has some limitations. We focus on healthy individuals without any diseases. Our study subjects are that the proportion of healthy and young people was high and the proportion of obese people was low. Therefore, our data cannot be generalized to subjects with any diseases. Also, the uncertain result of liver-related parameters, such as ALT, AST, and GGT levels, might be due to the small sample size and selection of the healthy subjects. In our study, the dropout rate was as high as 30% or more, which was due to reasons such as refusal to revisit due to the COVID-19 situation, personal reasons, and lack of compliance.

However, to our knowledge, this study is the first double-blind randomized controlled trial to examine liver-related parameters and lipid profile levels after FRVE intervention in humans. This study possesses several study strengths; it elucidates the efficacy of FRVE in TG and IR improvement and reports safety concerns about FRVE consumption because there were no reports of adverse effects during the 12-week intervention in 52 subjects.

In a review study, nutrients such as whole grains, polyunsaturated fatty acids, omega 3-fatty acids, vegetable proteins, and probiotics can help prevent and treat NAFLD, whereas excessive carbohydrates, simple sugars, saturated fats, trans-fatty acids, animal proteins, processed foods, and low dietary fiber intake are associated with an increase in NAFLD [4]. Therefore, as nutritional factors are very important indicators for the prevention of NAFLD, it is necessary to investigate the effects of combination with other dietary factors or at different doses of FRVE to prevent NAFLD in future studies.

## Conclusions

In conclusion, no significant results could be confirmed with respect to the liver-related parameters and serum lipid profiles in our study.

## Supplementary Information

**Additional file 1.** CONSORT 2010 Flow Diagram

## Data Availability

The datasets generated and/or analyzed during the current study are not publicly available due to privacy of subjects, but are available from the corresponding author on reasonable request**.**

## Abbreviations

AMPK: AMP-activated protein kinase
ALT: Alanine aminotransferase
AST: Aspartate aminotransferase
ALP: Alkaline phosphatase
BMI: Body mass index
BUN: Blood urea nitrogen
BP: Blood pressure
CBC: Complete blood count
DBP: Diastolic blood pressure
FRV: Fermented *Rhus verniciflua* Stokes
FRVE: Fermented *Rhus verniciflua* Stokes extract
FFA: Free fatty acids
GGT: Glutamyl transferase
HDL-C: High-density lipoprotein cholesterol
Hct: Hematocrit
Hb: Hemoglobin
HOMA-IR: Homeostasis model assessment
IR: Insulin resistance
LDL-C: Low-density lipoprotein cholesterol
MDA: Malondialdehyde
NAFLD: Nonalcoholic fatty liver disease
PLT: Platelet
RVSE: *Rhus verniciflua* Stokes extract
RVS: *Rhus verniciflua* Stokes
RBC: Red blood cells
SBP: Systolic blood pressure
SPSS: Statistical Package for the Social Sciences
TC: Total cholesterol
TG: Triglycerides
WBC: White blood cells
