# Supplementary material for: Effect of fermented Rhus verniciflua stokes extract on liver function parameters in healthy Korean adults: a double-blind randomized controlled trial
Source: Trials. 2021 Nov 22;22:830. doi: 10.1186/s13063-021-05656-0 (PMC8607399; doi:10.1186/s13063-021-05656-0)
Supplement: Supplementary file 1 — Additional file 1. CONSORT 2010 Flow Diagram [file 13063_2021_5656_MOESM1_ESM.doc]

**
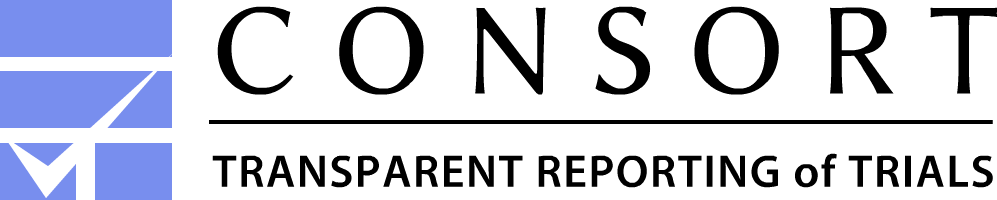
**

**CONSORT 2010 Flow Diagram**

**Allocation**

**Analysis**

**Follow-Up**

**Enrollment**

Assessed for eligibility (n=79)

Analysed ( n= 27 )
♦ Excluded from analysis (suboptimal compliance) ( n=6 )

Lost to follow-up (personal reasons) ( n= 3 )

Lost to follow-up (refusal to revisit) ( n= 3 )

Allocated to intervention ( n= 39 )

♦ Received allocated intervention ( n= 39 )

♦ Did not receive allocated intervention ( n=0 )

Lost to follow-up (personal reasons) ( n= 2 )

Lost to follow-up (refusal to revisit) ( n= 7 )

Allocated to intervention ( n= 40 )

♦ Received allocated intervention ( n= 40 )

♦ Did not receive allocated intervention ( n=0 )

Analysed ( n= 25 )
♦ Excluded from analysis (suboptimal compliance) ( n=6 )

Randomized (n=79)
